# Adherence to Healthy Lifestyle and Attenuation of Biological Aging in Middle-Aged and Older Chinese Adults

**DOI:** 10.1093/gerona/glab213

**Published:** 2021-07-30

**Authors:** Junning Fan, Canqing Yu, Yuanjie Pang, Yu Guo, Pei Pei, Zhijia Sun, Ling Yang, Yiping Chen, Huaidong Du, Dianjianyi Sun, Yanjie Li, Junshi Chen, Robert Clarke, Zhengming Chen, Jun Lv, Liming Li, China Kadoorie Biobank Collaborative Group

**Affiliations:** 1 Department of Epidemiology and Biostatistics, School of Public Health, Peking University, Beijing, China; 2 Peking University Center for Public Health and Epidemic Preparedness & Response, Beijing, China; 3 Chinese Academy of Medical Sciences, Beijing, China; 4 Medical Research Council Population Health Research Unit at the University of Oxford, UK; 5 Clinical Trial Service Unit and Epidemiological Studies Unit (CTSU), Nuffield Department of Population Health, University of Oxford, UK; 6 NCDs Prevention and Control Department, Nangang CDC, Heilongjiang, China; 7 China National Center for Food Safety Risk Assessment, Beijing, China; 8 Key Laboratory of Molecular Cardiovascular Sciences (Peking University), Ministry of Education, Beijing, China

**Keywords:** Epidemiology, Frailty, Lifestyle factor, Longevity

## Abstract

**Background:**

Little is known about the effects of lifestyle modification on biological aging in population-based studies of middle-aged and older adults.

**Method:**

We examined the individual and joint associations of multiple lifestyle factors with accelerated biological aging measured by change in frailty index (FI) over 8 years in a prospective study of Chinese adults. Data were obtained on 24 813 participants in the China Kadoorie Biobank on lifestyle factors and frailty status at baseline and at 8 years after baseline. Adherence to healthy lifestyle factors included nonsmoking or quitting smoking for reasons other than illness, avoidance of heavy alcohol consumption, daily intake of fruit and vegetables, being physically active, body mass index of 18.5–23.9 kg/m^2^, and waist-to-hip ratio of <0.90 (men)/0.85 (women). FI was constructed separately at baseline and resurvey using 25 age- and health-related items.

**Results:**

Overall, 8 760 (35.3%) individuals had a worsening frailty status. In multivariable-adjusted logistic regression analyses, adherence to healthy lifestyle was associated with a lower risk of worsening frailty status. Compared with robust participants maintaining 0–1 healthy lifestyle factors, the corresponding odds ratios (95% CIs) were 0.93 (0.83–1.03), 0.75 (0.67–0.84), 0.68 (0.60–0.77), and 0.55 (0.46–0.65) for robust participants with 2, 3, 4, and 5–6 healthy lifestyle factors. The decreased risk of frailty status worsening by adherence to healthy lifestyle factors was similar in both middle-aged and older adults, and in both robust and prefrail participants at baseline.

**Conclusions:**

Adherence to a healthy lifestyle may attenuate the rate of change in biological aging in middle-aged and older Chinese adults.

Many lifestyle factors are established risk factors for major chronic diseases and premature death in both Western and Chinese populations (1–5). In recent years, the relevance of adherence to healthy lifestyle factors has also been extended to include a decline in biological aging. Accelerated aging is a phenomenon in which individuals experience a faster rate of change in aging compared with age-matched controls (6). Accelerated aging commences in early adult life, hence, identifying strategies to attenuate the rate of change of biological aging could have substantial public health significance.

Several studies conducted in Western populations have reported that lifestyle factors were associated with accelerated biological aging assessed by DNA methylation (7,8) or telomere length (9). However, such molecular biomarkers of biological aging are expensive and difficult to measure and, consequently, are rarely available in large population-based studies of the determinants of accelerated aging. In contrast, frailty index (FI) is an indicator of biological age that is simple to measure and is widely available (10). Previous reports, including our recent publication, have demonstrated that FI predicts risks of all-cause mortality compared with healthy age-matched individuals (11,12). Moreover, the FI, together with measures of DNA methylation, had strong and independent effects on risks of mortality compared with other measures of biological aging (13).

Previous studies of European and U.S. populations have examined the associations of lifestyle factors with FI as a proxy for biological aging (14–16). Most previous studies of frailty have been restricted to individuals older than 50 years, but whether lifestyle modification can attenuate the rate of change of biological aging, especially before old age, requires further studies. Moreover, previous studies assessed the effects of lifestyle factors that were recorded on a single occasion at baseline, and did not consider the effects of changes in lifestyle factors over time.

The present study investigated the individual and joint associations of multiple lifestyle factors with accelerated biological aging measured by change in FI over about 8 years in middle-aged and older Chinese adults. In addition, we compared these associations in both robust and prefrail participants at baseline. We hypothesized that adherence to healthy lifestyle factors may lower the risk of frailty status worsening.

## Method

### Study Design and Participants

We used data from the China Kadoorie Biobank (CKB), a large prospective cohort study of 500 000 participants, aged 30–79 years, who were enrolled in 2004–2008 and followed up until the present time (17). Immediately after the baseline survey, the first resurvey was done during August and October 2008, when a random sample of 5% of surviving participants from the 10 study areas were included. The second resurvey took place in 2013–2014. The priority was given to those who participated in the first resurvey, supplemented by some new samples using cluster random sampling approach, of which the administrative unit (ie, rural village or urban residential committee) was used as the primary sampling unit. A total of 25 041 participants completed the second resurvey. The information collected at the resurvey was comparable to that collected at baseline, and also included several new additional questions and items for physical examination (more detailed information of questionnaires can be found at https://www.ckbiobank.org/site/Study+Resources). Ethics approval was obtained from the Oxford University Tropical Research Ethics Committee and the Chinese Center for Disease Control and Prevention Ethical Review Committee. All participants provided written informed consent.

In this study, only participants attending both baseline and the second resurvey and without missing values on body mass index (BMI) were included (*n* = 25 040). Frail participants at baseline were more likely to be survivors and change their lifestyle after the development of frailty, and, hence they were not included in the present analyses, leaving 24 813 baseline robust and prefrail participants in the primary analysis.

### Construction of FI

The procedure for constructing an FI in the CKB population followed a standard rule (10) and was detailed in our previous publication (12). Briefly, deficits were considered to be eligible for inclusion in the FI if they (i) were age- and health-related, (ii) had a baseline prevalence of ≥0.5%, (iii) had a prevalence <80% at age 50 years, (iv) comprised multiple body systems, and (v) had a missingness rate of ≤5% in the population. After screening all available data in CKB, we selected 28 variables to construct the FI, including diseases (eg, diabetes, hypertension), symptoms (eg, body pain, cough), physical measurements (eg, heart rate), and general health status. Each variable was coded as 0–1, with 0 indicating deficit absence and 1 indicating deficit presence. In the present study, 3 items (ie, physical activity, BMI, and waist-to-hip ratio [WHR]) that constitute the FI were also exposures in the analyses, and, hence, were removed from the FI, leaving 25 items in the FI (Supplementary Table S1). FI was calculated as the number of deficits present in a person divided by the total number of deficits included (ie, *n* = 25). We divided the continuous FI into 3 groups: robust (FI ≤ 0.10), prefrail (0.10 < FI < 0.25), and frail (FI ≥ 0.25) (18–20).

The FIs for baseline and the second resurvey were created separately. At baseline, all items that constituted the FI were either self-reported or measured variables. At the second resurvey, the variables that constituted the FI were the same as that measured at baseline except for diseases. To compensate for the possible underreporting of diseases, the disease status of participants was additionally supplemented by the occurrence of disease that was ascertained during the follow-up period between baseline and the second resurvey. The long-term follow-up for cause-specific morbidity and mortality was achieved through electronic linkage, via unique national ID number, to death and disease registries and health insurance databases. The International Classification of Diseases, 10th revision, codes used to define these diseases recorded during follow-up are outlined in Supplementary Table S2.

### Assessment of Frailty Transitions

Frailty transitions were defined according to change in frailty status between baseline and the second resurvey, with robust remained, robust worsening (from robust to prefrail or frail), prefrail remained, prefrail regression (from prefrail to robust), and prefrail worsening (from prefrail to frail). In the primary analysis, considering the limited number among prefrail participants, we combined prefrail remained and prefrail regression into one group of prefrail remained or regression. In the secondary analysis, we also assessed the association of lifestyle factors with prefrail regression. The mean FI value change per 5 years was calculated as the FI value at the second resurvey minus the FI value at baseline, divided by the time interval between these 2 surveys and multiplied by 5 years.

### Assessment of Lifestyle Factors and Covariates

Information about lifestyle factors or other covariates were obtained through an interviewer-administered laptop-based questionnaire at both surveys. For smoking status, we asked current smoking status for all participants; the frequency, type, and amount of tobacco smoked per day for ever smokers; and years after quitting and reasons for quitting for former smokers. For alcohol consumption, we asked the usual frequency, type of alcoholic beverage drank habitually, and volume drank on a usual drinking day in the past 12 months. For dietary intake frequency, a short qualitative food frequency questionnaire, with reasonably good validity and reproducibility, was used to assess habitual intakes of 12 major food groups (ie, rice, wheat, other staple food, meat, poultry, fish or sea food, eggs, fresh vegetables, soybean products, preserved vegetables, fresh fruit, and dairy products) in the past 12 months (2). The possible answers were daily, 4–6 d/wk, 1–3 d/wk, monthly, or never/rarely. For physical activity, participants were asked about their usual type and duration of activities spent on work, commuting, housework, or leisure time-related domains in the past 12 months. Total physical activity level was calculated by multiplying the metabolic equivalent tasks (METs) value for each activity by hours spent on that activity per day and summing the MET-hours for all activities. Covariates information includes sociodemographic characteristics (age, sex, geographic location, and education) and personal medical history.

Physical measurements, including height, weight, and waist and hip circumference, were undertaken by trained staff. BMI was calculated as weight (kg) divided by the square of standing height (m). The WHR was the ratio of waist circumference to hip circumference.

### Definition of Healthy Lifestyle Factors

Consistent with previous studies (1–3), 6 healthy lifestyle factors were defined as follows. For smoking, the healthy group was defined as never smoking or having stopped for reasons other than illness. For alcohol consumption, the healthy group was those less than weekly drinkers, weekly but not daily drinkers, or daily drinkers with an intake of <30 g of pure alcohol in men or <15 g in women. For dietary habits, eating both fruit and vegetables daily was considered to be healthy (21,22). For physical activity, the healthy group referred to those being sex-specific upper 50% of the physical activity level. We also used 2 measures of body composition to define healthy group if they had BMI of 18.5–23.9 kg/m^2^ (23) and WHR <0.90 in men or <0.85 in women (24), respectively. For each kind of lifestyle factor, participants were classified into 3 states according to their lifestyle change between baseline and the second resurvey: maintaining a constant healthy lifestyle, fluctuating lifestyle (including desirable transition, ie, transition from unhealthy lifestyle to healthy lifestyle; and undesirable transition, ie, transition from healthy lifestyle to unhealthy lifestyle), and maintaining an unhealthy lifestyle.

### Statistical Analysis

According to frailty status transitions, baseline characteristics of participants were presented as means for continuous variables or percentages (%) for categorical variables. Given that baseline health status may affect lifestyle and frailty transitions, all analyses were conducted in baseline robust and prefrail participants separately. Mean FI value at the second resurvey and FI value change per 5 years were calculated by baseline lifestyle factors or lifestyle changes from baseline to the second resurvey, adjusting for age, sex, and 10 study areas.

Logistic regression analysis was used to examine the association of frailty status transitions with baseline lifestyle factors or lifestyle changes from baseline to the second resurvey. Multivariable models included age, sex, 10 study areas, education, and 6 lifestyle factors (smoking status, alcohol consumption, intake of fruit and vegetables, physical activity, BMI, and WHR). Stratified analyses by baseline age (younger than 60 years and 60 years and older) and sex (men, women) were performed only in the baseline robust participants because the number of participants in the baseline prefrail group was limited. Sensitivity analyses were performed by (i) additionally adjusting for the time interval between baseline and the second resurvey and (ii) not including follow-up information to supplement disease status at the second resurvey.

To assess the individual association of each constant healthy lifestyle with frailty status transitions, we included all the dichotomized healthy lifestyle change variables as independent variables, with the constant unhealthy and fluctuating status group as the reference group. The cumulative effect of constant healthy lifestyle on frailty transitions was examined by putting the number of constant healthy lifestyle factors as a categorical variable in the regression model. A test for linear trend test was performed by setting the number of constant healthy lifestyle factors as a continuous variable in the model. All these analyses were performed using logistic regression, adjusting for age, sex, 10 study areas, and education. Stratified analyses by age at baseline were also conducted, as mentioned before.

All of the analyses were performed using Stata version 15.0 (StataCorp LP, College Station, TX), and R version 3.5.3 was used to graph results. Statistical significance was set at a 2-tailed *p* <.05.

## Results

The 28-item FI was significantly associated with mortality in the overall CKB population (12). In the present subset of CKB population, at both baseline and the second resurvey, the mean FI and prevalence of frailty in the study participants increased with age; women had a higher level of frailty than men (Supplementary Figure S1). The FI was predictive of total mortality (Supplementary Table S3), providing support for the validity of the FI in the present study.

### Characteristics of the Study Participants

The median interval from baseline to the second resurvey was 8.0 (interquartile range: 5.5–9.7) years. Among the robust participants at baseline, 55.2% remained robust, 42.0% progressed from robust to prefrail, and 2.8% progressed from robust to frail (Supplementary Table S4). Participants with robust worsening were more likely to be older, have lower education level, be daily smokers or heavy drinkers (only for men), and less likely to eat fruit and vegetables daily. They also tend to have higher BMI or WHR, and have higher prevalence of medical histories (Table 1). Among the prefrail participants at baseline, 88.2% remained prefrail or regressed to robust, and 11.8% progressed to frail status. Compared with participants remaining or improving in baseline frailty status, those worsening in frailty status were more likely to be older, female, and live in the urban areas. They were also more likely to adopt an unhealthy lifestyle and have chronic diseases at baseline.

**Table 1.** Baseline Characteristics of the Study Participants by Frailty Status Transitions From Baseline to the Second Resurvey

	Baseline Robust		Baseline Prefrail	
	Remained	Worsening	Remained or Regression	Worsening
*N* (%)	9 756 (39.3)	7 916 (31.9)	6 297 (25.4)	844 (3.4)
Sociodemographic factors				
Age, year (*SD*)	47.8 (9.1)	52.6 (10.0)	54.6 (10.0)	59.1 (8.5)
Women, %	61.0	61.3	62.4	67.8
Urban area, %	43.3	44.6	40.1	50.5
Middle school and higher, %	49.2	45.8	46.7	46.0
Lifestyle factors				
Male participants: daily smoking^a^, %	64.5	70.2	69.1	70.6
Female participants: daily smoking, %	2.8	2.2	2.6	3.6
Male participants: heavy alcohol drinking^b^, %	22.0	25.4	25.3	23.6
Female participants: heavy alcohol drinking, %	2.2	1.9	2.3	2.5
Eating fruit and vegetables daily, %	18.8	16.1	16.3	14.5
Physical activity, MET-h/d	21.9	21.7	20.7	20.6
BMI < 18.5 kg/m^2^, %	3.8	3.7	4.2	3.2
BMI ≥ 24.0 kg/m^2^, %	40.5	47.0	46.3	52.4
WHR ≥ 0.90 (men) or 0.85 (women), %	52.1	58.8	58.4	63.3
Medical history, %				
Hypertension	21.3	31.7	53.3	56.0
Heart disease	0.6	1.1	5.6	8.6
Stroke or transient ischemic attack	0.2	0.3	2.2	2.3
Emphysema or bronchitis	0.4	0.9	5.3	11.6
Tuberculosis	0.2	0.7	2.3	2.4
Asthma	0.1	0.2	0.9	3.5
Peptic ulcer	2.0	2.3	8.9	8.1
Gallstone diseases	2.2	4.2	11.6	13.0
Rheumatoid arthritis	0.8	1.1	5.0	6.2
Fracture	3.8	4.8	13.9	14.4
Neurasthenia	0.2	0.4	3.2	4.8
Diabetes	1.0	2.8	10.0	14.8
Cancer	0.2	0.1	0.8	1.8
Chronic kidney disease	0.5	0.8	2.6	2.3

*Notes*: BMI = body mass index; MET = metabolic equivalent task; WHR = waist-to-hip ratio. Baseline characteristics were adjusted for age, sex, and 10 study area, except for these 3 variables.

^a^Those who stopped smoking due to illness were classified as daily smoking.

^b^Heavy alcohol drinking refers to current drinking ≥30 g/d of pure alcohol in men or ≥15 g/d in women or ex-drinkers.

### FI and Its Change per 5 Years According to Lifestyle Characteristics

Among baseline robust and prefrail participants, the average FI value changes per 5 years were 0.034 and 0.009; the mean FI values at the second resurvey were 0.095 and 0.151, respectively (Supplementary Table S5). Participants with the following lifestyle characteristics at baseline showed a higher increase in the FI values per 5 years and higher FI value at the second resurvey: ever smoking, former or heavy alcohol drinking, consuming fruit and vegetables less than daily, lower level of physical activity, and being generally or abdominally obese. When considering lifestyle changes between baseline and the second resurvey, those who maintained a constant healthy lifestyle had the lowest FI values at the second resurvey (Supplementary Table S6). In contrast, those maintaining an unhealthy lifestyle had the highest FI values. When compared with participants with undesirable transition, generally, those with desirable transition had lower increase in the FI values per 5 years and lower FI values at the second resurvey.

### Lifestyle Characteristics and Frailty Transitions

For robust participants, the baseline lifestyle characteristics that were associated with worsening in frailty status included moderate (odds ratio [OR] = 1.25 [95% CI: 1.10–1.42]) or heavy smoking (1.47 [1.24–1.74]), consuming fruit and vegetables less than daily (1.23 [1.12–1.35]), BMI ≥24.0 kg/m^2^ (24.0–27.9 kg/m^2^: 1.13 [1.05–1.22]; ≥28.0 kg/m^2^: 1.54 [1.37–1.74]), and WHR ≥0.90 (men)/0.85(women) (0.90–0.94 [men]/0.85–0.89 [women]: 1.13 [1.04–1.22]; ≥0.95 [men]/0.90 [women]: 1.35 [1.23–1.49]) (Table 2). These associations were consistently observed in participants younger than 60 years. While in participants aged 60 years and older, only daily smoking and BMI ≥24.0 kg/m^2^ were associated with an increased risk of worsening frailty status. For men, daily smoking, former drinking, eating fruit and vegetables less than daily, BMI ≥24.0 kg/m^2^, and WHR ≥0.90 had increased risk of worsening frailty status (Supplementary Table S7). While for women, eating fruit and vegetables less than daily, BMI ≥24.0 kg/m^2^, and WHR ≥0.85 had increased risk. For prefrail participants, former smoking or daily smoking of 15–24 cigarettes or equivalent was associated with increased risk of worsening frailty status.

**Table 2.** Association Between Baseline Lifestyle Factors and Frailty Transitions From Baseline to the Second Resurvey

	Robust Worsening						Prefrail Worsening	
	Total		<60 y		≥60 y			
	Cases	OR (95% CI)	Cases	OR (95% CI)	Cases	OR (95% CI)	Cases	OR (95% CI)
Smoking status								
Nonsmoking	5 470	1.00	4 270	1.00	1 200	1.00	600	1.00
Former smoker^a^	225	1.06 (0.86–1.31)	123	1.03 (0.79–1.33)	102	1.25 (0.85–1.83)	30	1.78 (1.12–2.85)
1–14 cig/d	775	1.10 (0.96–1.25)	489	1.01 (0.86–1.18)	286	1.42 (1.08–1.87)	91	1.32 (0.98–1.78)
15–24 cig/d	1 003	1.25 (1.10–1.42)	766	1.16 (1.01–1.34)	237	1.66 (1.22–2.24)	84	1.41 (1.02–1.95)
≥25 cig/d	443	1.47 (1.24–1.74)	345	1.35 (1.12–1.63)	98	1.90 (1.24–2.90)	39	1.48 (0.97–2.25)
Alcohol consumption								
Nondrinking	6 494	1.00	4 956	1.00	1 538	1.00	717	1.00
Former drinker	263	1.14 (0.94–1.38)	159	1.07 (0.86–1.34)	104	1.28 (0.87–1.89)	46	0.94 (0.66–1.33)
Weekly or <15/30 g/d^b^	613	0.92 (0.81–1.04)	485	0.98 (0.86–1.13)	128	0.70 (0.52–0.94)	49	0.84 (0.60–1.17)
≥15/30 g/d	546	1.02 (0.89–1.17)	393	1.09 (0.93–1.27)	153	0.76 (0.56–1.04)	32	0.77 (0.51–1.16)
Eating fruit and vegetables								
Daily	1 312	1.00	968	1.00	344	1.00	155	1.00
Less than daily	6 604	1.23 (1.12–1.35)	5 025	1.22 (1.10–1.36)	1 579	1.23 (0.97–1.56)	689	1.24 (0.99–1.54)
Physical activity (MET-h/d)								
Quartile 1 (lowest)	1 945	1.09 (0.99–1.21)	1 133	1.10 (0.98–1.23)	812	0.94 (0.70–1.26)	335	1.05 (0.81–1.37)
Q2	2 083	1.02 (0.93–1.13)	1 495	1.06 (0.96–1.17)	588	0.81 (0.61–1.09)	227	0.93 (0.71–1.21)
Q3	1 968	1.02 (0.93–1.11)	1 653	1.03 (0.93–1.12)	315	0.93 (0.69–1.25)	170	1.10 (0.85–1.42)
Q4 (highest)	1 920	1.00	1 712	1.00	208	1.00	112	1.00
BMI (kg/m^2^)								
<18.5	317	1.15 (0.97–1.36)	208	1.20 (0.99–1.45)	109	1.01 (0.71–1.43)	30	0.87 (0.58–1.30)
18.5–23.9	3 907	1.00	2 929	1.00	978	1.00	354	1.00
24.0–27.9	2 724	1.13 (1.05–1.22)	2 107	1.12 (1.03–1.22)	617	1.21 (1.00–1.48)	317	1.19 (0.99–1.42)
≥28.0	968	1.54 (1.37–1.74)	749	1.52 (1.34–1.74)	219	1.67 (1.22–2.29)	143	1.26 (0.98–1.61)
WHR								
Men < 0.90, women < 0.85	3 269	1.00	2 534	1.00	735	1.00	278	1.00
Men 0.90–0.94, women 0.85–0.89	2 517	1.13 (1.04–1.22)	1 935	1.13 (1.04–1.23)	582	1.14 (0.94–1.40)	247	0.99 (0.81–1.21)
Men ≥ 0.95, women ≥ 0.90	2 130	1.35 (1.23–1.49)	1 524	1.39 (1.25–1.54)	606	1.24 (0.99–1.54)	319	1.21 (0.99–1.49)

*Notes*: BMI = body mass index; MET = metabolic equivalent task; WHR = waist-to-hip ratio. Multivariable model was adjusted for age, sex, 10 study area, education, and lifestyle factors listed in the table. In the analysis of robust worsening, those remained robust were set as the reference group; in the analysis of prefrail worsening, those remained or improved were set as the reference group. All the *p* values for interaction between lifestyle factors and age were >.05.

^a^Former smoker refers to those who stopped smoking for reasons other than illness. Those who stopped smoking due to illness were classified as daily smoker.

^b^Drinking <30 g/d of pure alcohol in men or <15 g/d in women.

Compared to robust participants with constant healthy lifestyle regarding smoking, eating fruit and vegetables, physical activity, BMI, and WHR, those maintaining an unhealthy lifestyle had 1.21-fold (95% CI: 1.08–1.35), 1.36-fold (1.21–1.53), 1.23-fold (1.12–1.35), 1.14-fold (1.05–1.23), and 1.27-fold (1.16–1.38) increased risk of worsening frailty status, respectively (Table 3). Similar results were found among participants younger than 60 years, but the effect of physical activity and BMI were no longer statistically significant among those aged 60 years and older. When stratified by sex, there was a statistical difference of the effect of BMI in men and women (*p*_interaction_ < .05), both constant unhealthy BMI and fluctuating status showed increased risk of robust worsening in men, while only constant unhealthy BMI had increased risk in women (Supplementary Table S8). Among baseline prefrail participants, fluctuating status of smoking and nondaily consumption of fruit and vegetables had higher risk of worsening prefrail status.

**Table 3.** Association of Lifestyle Changes With Frailty Transitions From Baseline to the Second Resurvey

	Robust Worsening						Prefrail Worsening	
	Total		<60 y		≥60 y			
	Cases	OR (95% CI)	Cases	OR (95% CI)	Cases	OR (95% CI)	Cases	OR (95% CI)
Smoking status								
Constant nonsmoking^a^	5 579	1.00	4 317	1.00	1 262	1.00	613	1.00
Fluctuating status	547	1.05 (0.90–1.21)	347	0.99 (0.83–1.17)	200	1.24 (0.93–1.66)	75	1.41 (1.03–1.93)
Constant smoking	1 790	1.21 (1.08–1.35)	1 329	1.14 (1.01–1.29)	461	1.50 (1.17–1.91)	156	1.23 (0.94–1.61)
Alcohol consumption								
Constant nonheavy alcohol drinking^b^	6 674	1.00	5 120	1.00	1 554	1.00	713	1.00
Fluctuating status	772	1.07 (0.95–1.20)	553	1.09 (0.96–1.24)	219	1.01 (0.78–1.31)	85	1.05 (0.80–1.37)
Constant heavy alcohol drinking	470	1.09 (0.94–1.26)	320	1.10 (0.93–1.30)	150	0.99 (0.72–1.36)	46	1.15 (0.81–1.64)
Eating fruit and vegetables								
Constant daily eating	949	1.00	699	1.00	250	1.00	111	1.00
Fluctuating status	2 124	1.30 (1.16–1.46)	1 654	1.27 (1.12–1.44)	470	1.46 (1.10–1.93)	223	1.30 (1.00–1.69)
Constant nondaily eating	4 843	1.36 (1.21–1.53)	3 640	1.36 (1.19–1.54)	1 203	1.35 (1.01–1.80)	510	1.34 (1.02–1.76)
Physical activity								
Constant being physically active^c^	2 337	1.00	2 162	1.00	175	1.00	127	1.00
Fluctuating status	2 859	1.10 (1.01–1.18)	2 312	1.09 (1.01–1.18)	547	1.18 (0.90–1.55)	287	1.12 (0.89–1.41)
Constant low physical activity	2 720	1.23 (1.12–1.35)	1 519	1.22 (1.10–1.35)	1 201	1.31 (0.99–1.72)	430	1.09 (0.85–1.40)
BMI (kg/m^2^)								
Constant healthy BMI^d^	2 986	1.00	2 236	1.00	750	1.00	262	1.00
Fluctuating status	1 518	1.04 (0.95–1.13)	1 134	1.03 (0.94–1.13)	384	1.05 (0.85–1.29)	163	1.09 (0.88–1.35)
Constant unhealthy BMI	3 412	1.14 (1.05–1.23)	2 623	1.14 (1.04–1.24)	789	1.18 (0.97–1.43)	419	1.17 (0.97–1.41)
WHR								
Constant healthy WHR^e^	1 803	1.00	1 367	1.00	436	1.00	151	1.00
Fluctuating status	2 061	1.04 (0.95–1.13)	1 587	1.03 (0.94–1.14)	474	1.08 (0.87–1.35)	200	1.04 (0.82–1.31)
Constant unhealthy WHR	4 052	1.27 (1.16–1.38)	3 039	1.27 (1.15–1.40)	1 013	1.31 (1.06–1.62)	493	1.19 (0.96–1.48)

*Notes*: BMI = body mass index; WHR = waist-to-hip ratio. Multivariable model was adjusted for age, sex, 10 study area, education, and lifestyle changes listed in the table. In the analysis of robust worsening, those remained robust were set as the reference group; in the analysis of prefrail worsening, those remained or improved were set as the reference group. All the *p* values for interaction between lifestyle changes and age were >.05.

^a^Nonsmoking refers to never smoking or having stopped for reasons other than illness.

^b^Heavy alcohol drinking refers to drinking ≥30 g/d of pure alcohol in men or ≥15 g/d in women or ex-drinkers.

^c^Being physically active refers to being sex-specific upper 50% of the physical activity level.

^d^Healthy BMI refers to 18.5–23.9 kg/m^2^.

^e^Healthy WHR refers to WHR <0.90 in men or <0.85 in women.

Results from sensitivity analyses showed that the ORs remained almost unchanged when adjusting for the time interval or not including follow-up information to supplement disease status at the second resurvey (Supplementary Tables S9 and S10). The analyses of lifestyle factors and prefrail status regression showed that BMI ≥28.0 kg/m^2^ and WHR ≥0.95 (men)/0.90 (women) at baseline, or constant with low physical activity, or constant being central obese, were less likely to regress to robust (Supplementary Tables S11 and S12).

### Healthy Lifestyle Factors and Frailty Transitions

**Figure 1.**
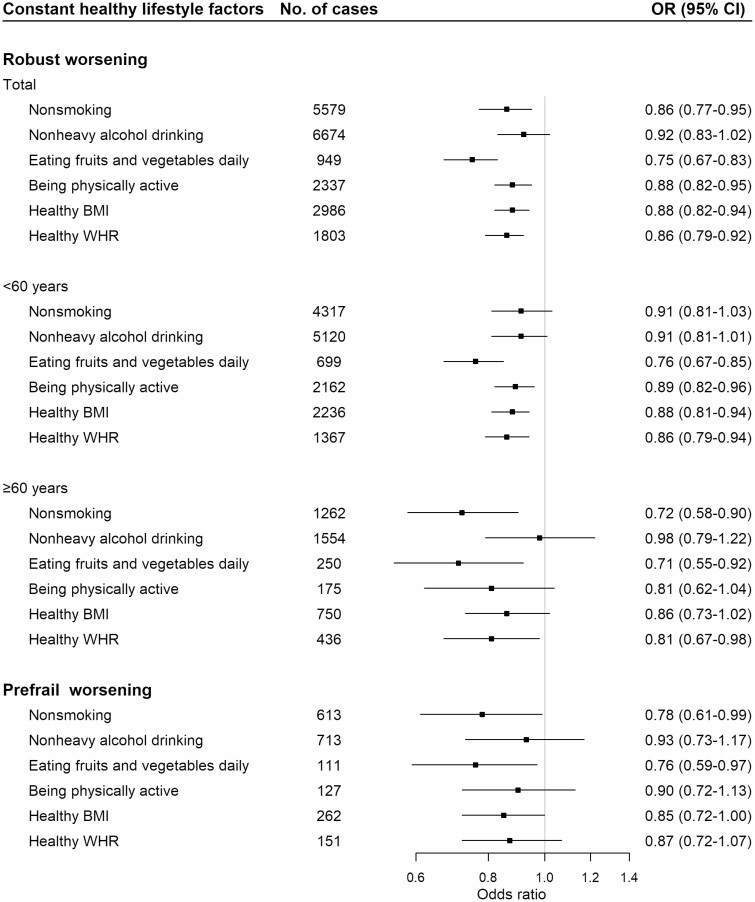
Association between constant healthy lifestyle factors and frailty transitions from baseline to the second resurvey. Multivariable model was adjusted for age, sex, 10 study areas, education, and constant healthy lifestyle factors listed in the figure, with the constant unhealthy and fluctuating status group as the reference. All the *p* values for interaction between constant healthy lifestyle factors and age were >.05. BMI = body mass index; OR = odds ratio; WHR = waist-to-hip ratio.

When we dichotomized these 6 healthy lifestyle change variables, constant nonsmoking, daily intake of fruit and vegetables, being physically active, keep healthy BMI, and healthy WHR in the total sample of robust participants were independently associated with 14% (OR = 0.86 [95% CI: 0.77–0.95]), 25% (0.75 [0.67–0.83]), 12% (0.88 [0.82–0.95]), 12% (0.88 [0.82–0.94]), and 14% (0.86 [0.79–0.92]) lower risk of worsening frailty status, while constant nonheavy alcohol consumption was not statistically significantly associated with robust worsening (Figure 1). The benefits of maintaining a healthy lifestyle were also observed in participants younger than 60 years except for constant nonsmoking and nonheavy alcohol drinking. While in participants aged 60 years and older, only constant nonsmoking, daily intake of fruit and vegetables, and keeping healthy WHR carried reduced risk of robust status worsening. Among baseline prefrail participants, constant nonsmoking and eating fruit and vegetables daily showed protective effects against prefrail status worsening.

**Figure 2.**
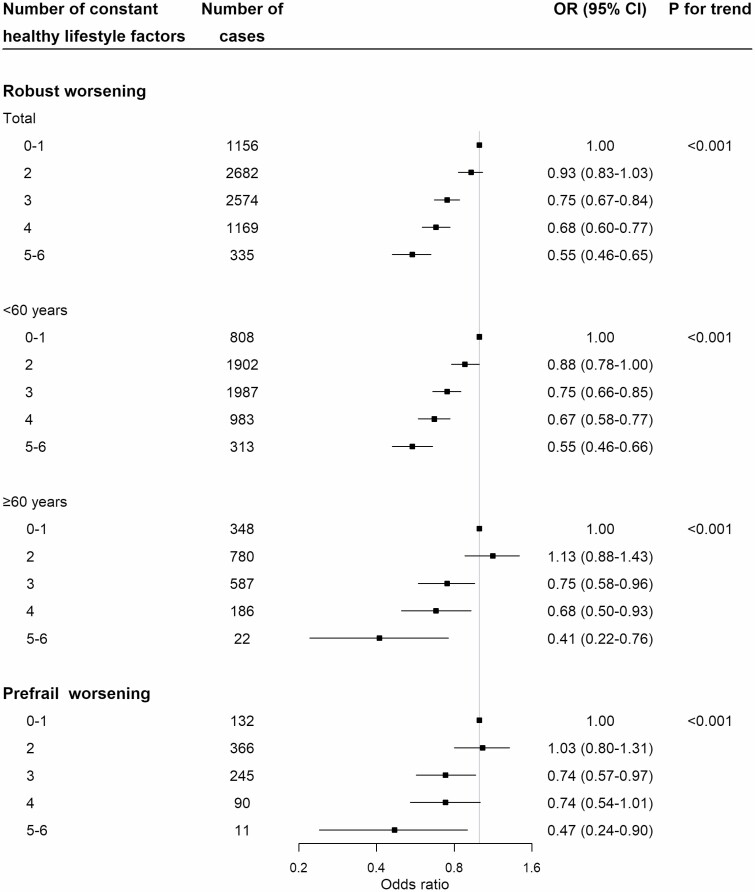
Number of constant healthy lifestyle factors and frailty transitions from baseline to the second resurvey. Constant healthy lifestyle factors from baseline to the second resurvey include nonsmoking, nonheavy alcohol drinking, daily intake of fruit and vegetables, being physically active, healthy body mass index, and healthy waist-to-hip ratio. Multivariable model was adjusted for age, sex, 10 study area, and education. The *p* value for interaction between number of constant healthy lifestyle factors and age were >.05. OR = odds ratio.

When the number of healthy lifestyle factors practiced by participants was treated as an ordinal scale (0–1, 2, 3, 4, 5–6), there was a statistically significant linear trend (*p*_trend_ < .001) with increasing number of healthy lifestyle factors associated with decreasing risk of worsening frailty status among robust and prefrail participants (Figure 2). Compared with robust participants with 0–1 constant healthy lifestyle factors, the corresponding ORs (95% CIs) were 0.93 (0.83–1.03), 0.75 (0.67–0.84), 0.68 (0.60–0.77), and 0.55 (0.46–0.65) for those with 2, 3, 4, and 5–6 healthy lifestyle factors. Similar results were observed among participants both aged younger than 60 years and 60 years and older, despite the cases of participants with 5–6 constant healthy lifestyle factors were small and the CIs were wide.

## Discussion

In the present study of about 25 000 Chinese adults, we used change in FI over an 8-year period as a surrogate measure for biological aging. Among robust participants at baseline, compared with age-similar peers, those with unhealthy lifestyle factors were more likely to have an accelerated biological aging, manifested by a higher risk of worsening frailty status. In contrast, participants who adhered to healthy lifestyles (ie, constant nonsmoking, eating fruit and vegetables daily, being physically active, and keeping BMI of 18.5–23.9 kg/m^2^, and WHR <0.90 in men or <0.85 in women) had a lower risk of worsening frailty status. Such health benefits on biological aging applied to both middle-aged and older adults and to both robust and prefrail individuals.

### Comparison With Previous Studies

Consistent with previous studies conducted chiefly in European countries (14–16,25–27), the findings of the present study demonstrated the adverse effects of unhealthy lifestyle factors on progression of frailty status in older people. The English Longitudinal Study of Ageing study of 8 780 participants aged 50 and older demonstrated that being a smoker or ex-smoker (hazard ratio [HR] = 1.29; 95% CI: 1.18–1.41), high BMI (HR = 1.33; 95% CI: 1.18–1.50), high WHR (HR = 1.25; 95% CI: 1.13–1.38), and low intensity of physical activity or sedentary behavior (HR = 2.17; 95% CI: 1.76–2.78) were all associated with increased risk of both frailty development and progression over 12-year follow-up (14). In the UK Whitehall II study of 6 357 adults, healthy behaviors at age 50, that is, nonsmoking (HR = 0.56; 95% CI: 0.44–0.71), moderate alcohol consumption (0.73; 0.61–0.88), ≥2.5 hours of physical activity per week (0.66; 0.54–0.81), and consumption of fruit and vegetables at least twice a week (0.76; 0.59–0.98) were associated with lower risk of incident frailty over 20.4 years’ follow-up (28). Hence, healthy lifestyle in middle age was related to the development of frailty in older age, but the present study extends such associations to the middle-aged adults.

The association of lifestyle factors with frailty status as a surrogate measure of biological aging is plausible. Similar findings have also been observed in studies using molecular biomarkers (eg, DNA methylation age (7,8), telomere length (9)) or other age-related changes in physical characteristics (29). The underlying mechanisms for the adverse effects of unhealthy lifestyle factors may include increased inflammation and oxidative stress (30–32) and reduced lean mass and muscle strength (33), which harm physical function and overall health status.

Lifestyle may change over time. However, there is little available evidence on the relationships between lifestyle changes and frailty transitions. The above-mentioned UK Whitehall II study found that compared to participants with 0–1 healthy behavior in 1985, those changed to 3–4 healthy behaviors in 1997 had 43% (HR = 0.57; 95% CI: 0.38–0.87) reduced subsequent risk of frailty (28). A prospective cohort of Koreans aged 65 years and older showed that participants following a healthy lifestyle or improving an unhealthy lifestyle over 2 years could help slow down the rate of cognitive decline (34). The present study demonstrated that adherence to healthy lifestyles over 8 years was associated with the lowest risks of worsening frailty status. Those maintaining 5 or 6 healthy lifestyle factors had a 45% lower risk of frailty progression, highlighting the importance of maintaining a healthy lifestyle over time.

An important finding of the present study was the association between healthy lifestyle factors and the progression of frailty by baseline frailty status. In contrast to the findings in robust participants at baseline, most associations between single constant healthy lifestyle factor and prefrail worsening were no longer statistically significant among prefrail worsening participants. One of the possible explanations may be due to the limited sample size and underpowering in prefrail participants, as the confidence interval in this group was wide and spanning 1. However, in the association of number of constant healthy lifestyle factors and prefrail worsening, there was a decreased risk of prefrail worsening with increased number of constant healthy lifestyle factors. These findings suggest that adherence to a healthy lifestyle for a prolonged period is an important determinant for prevention of worsening frailty status in both baseline robust and prefrail participants. In addition, not all constant healthy lifestyle factors were protective for all age groups, the impact of physical activity and BMI lost statistical significance among those aged 60 years and older, manifested as wide confidence interval, possibly due to underpowering caused by small cases.

### Strengths and Limitations

The present study is the first study in the Chinese population to assess the individual and joint associations of lifestyle factors with accelerated aging measured by change in FI. The strength of the present study includes its prospective design and comprehensive information on multiple lifestyle factors and health states related to multiple body systems. Access to a large sample size and a wide range of age groups (30–79 years) enabled the study to conduct analyses by age-specific groups and baseline frailty status. The exposure factors not only included lifestyle factors at baseline but also included changes in lifestyle over an 8-year follow-up in contrast to previous studies that were restricted to lifestyle measures at baseline.

Our study also had several limitations. First, the deficits that constituted the FI are mostly being medical histories and symptoms, with a lack of functional measures like activities of daily living or instrumental activities of daily living. We also removed physical activity, BMI, and WHR from the 28-item FI we constructed before (12). Nevertheless, the FI is not sensitive to specific items and has been proved to be valid and robust in a previous study (35). Second, lifestyle measurements at 2 timepoints of baseline and resurvey may not capture the fluctuations during the period. Also, some information on medical history and lifestyle factors were self-reported. These potential information biases are more likely to be nondifferential and attenuate the effect size toward the null. Third, for foods such as fruit and vegetables, we only collected information on frequency but not on specific portion sizes. We cannot calculate caloric intake either. Fourth, the number of participants in prefrail status was limited, potentially leading to some underpowered analyses. Fifth, the participants included in the present analysis were those who survived to attend the second resurvey and possible to be healthier survivors, which may underestimate the effect of healthy lifestyle factors. Lastly, the CKB was not designed to be representative of the whole population in China. Considering the diversity of lifestyles and risk exposures in different populations, we should be cautious when generalizing the conclusions to other populations.

## Conclusion

In this prospective study of middle-aged and older Chinese adults, adherence to healthy lifestyles (ie, nonsmoking, nonheavy alcohol drinking, daily consumption of fruit and vegetables, being physically active, and maintenance of a healthy weight and body shape) was associated with lower risk of accelerated biological aging. The present study added to the existing evidence that adherence to a healthy lifestyle is particularly important for slowing down the pace of accelerated aging in otherwise robust middle-aged adults.

## Funding

This work was supported by the National Natural Science Foundation of China (81941018). The China Kadoorie Biobank baseline survey and the first resurvey were supported by a grant from the Kadoorie Charitable Foundation in Hong Kong. The long-term follow-up is supported by grants from the National Key R&D Program of China (2016YFC0900500), the National Natural Science Foundation of China (81390540), and the Chinese Ministry of Science and Technology (2011BAI09B01). The funders had no role in the study design, data collection, data analysis and interpretation, writing of the report, or the decision to submit the article for publication.

## Supplementary Material

glab213_suppl_Supplementary_Materials

## Data Availability

The access policy and procedures are available at www.ckbiobank.org.
