# Supplementary material for: Adherence to Healthy Lifestyle and Attenuation of Biological Aging in Middle-Aged and Older Chinese Adults
Source: J Gerontol A Biol Sci Med Sci. 2021 Jul 30;76(12):2232–41. doi: 10.1093/gerona/glab213 (PMC8599067; doi:10.1093/gerona/glab213)
Supplement: glab213_suppl_Supplementary_Materials [file glab213_suppl_supplementary_materials.pdf]

.

Supplementary material

Adherence to healthy lifestyle and attenuation of biological aging in middle-aged and older Chinese adults

Appendix Members of the China Kadoorie Biobank collaborative group

Table S1 List of 25 variables included in the FI

Table S2 ICD-10 codes used to define the occurrence of disease from baseline to the 2nd resurvey

Figure S1 Mean FI and frailty prevalence by age and sex among 25,040 participants

Table S3 Association between FI and total mortality among 25,040 participants

Table S4 Frailty transitions from baseline to the 2nd resurvey

Table S5 Mean FI value change per 5 years and mean FI value at the 2nd resurvey by baseline frailty status and lifestyle factors

Table S6 Mean FI value change per 5 years and mean FI value at the 2nd resurvey by baseline frailty status and lifestyle change between baseline and the 2nd resurvey

Table S7 Association between baseline lifestyle factors and frailty transitions from baseline to the 2nd resurvey among baseline robust participants by gender

Table S8 Association of lifestyle changes with frailty transitions from baseline to the 2nd resurvey among baseline robust participants by gender

Table S9 Association between baseline lifestyle factors and frailty transitions by not including follow-up information to supplement diseases status at the second resurvey

Table S10 Association between lifestyle changes and frailty transitions by not including follow-up information to supplement diseases status at the second resurvey

Table S11 Association of baseline lifestyle factors with prefrail regression and prefrail worsening

Table S12 Association of lifestyle changes with prefrail regression and prefrail worsening

Appendix Members of the China Kadoorie Biobank collaborative group

International Steering Committee: Junshi Chen, Zhengming Chen (PI), Robert Clarke, Rory Collins, Yu Guo, Liming Li (PI), Jun Lv, Richard Peto, Robin Walters. **International Co-ordinating Centre, Oxford:** Daniel Avery, Ruth Boxall, Derrick Bennett, Yumei Chang, Yiping Chen, Zhengming Chen, Robert Clarke, Huaidong Du, Simon Gilbert, Alex Hacker, Mike Hill, Michael Holmes, Andri Iona, Christiana Kartsonaki, Rene Kerosi, Ling Kong, Om Kurmi, Garry Lancaster, Sarah Lewington, Kuang Lin, John McDonnell, Iona Millwood, Qunhua Nie, Jayakrishnan Radhakrishnan, Paul Ryder, Sam Sansome, Dan Schmidt, Paul Sherliker, Rajani Sohoni, Becky Stevens, Iain Turnbull, Robin Walters, Jenny Wang, Lin Wang, Neil Wright, Ling Yang, Xiaoming Yang. **National Co-ordinating Centre, Beijing:** Zheng Bian, Yu Guo, Xiao Han, Can Hou, Jun Lv, Pei Pei, Chao Liu, Canqing Yu. **10 Regional Co-ordinating Centres: Qingdao CDC:** Zengchang Pang, Ruqin Gao, Shanpeng Li, Shaojie Wang, Yongmei Liu, Ranran Du, Yajing Zang, Liang Cheng, Xiaocao Tian, Hua Zhang, Yaoming Zhai, Feng Ning, Xiaohui Sun, Feifei Li. **Licang CDC:** Silu Lv, Junzheng Wang, Wei Hou. **Heilongjiang Provincial CDC:** Mingyuan Zeng, Ge Jiang, Xue Zhou. **Nangang CDC:** Liqiu Yang, Hui He, Bo Yu, Yanjie Li, Qinai Xu,Quan Kang, Ziyan Guo. **Hainan Provincial CDC:** Dan Wang, Ximin Hu, Jinyan Chen, Yan Fu, Zhenwang Fu, Xiaohuan Wang. **Meilan CDC:** Min Weng, Zhendong Guo, Shukuan Wu,Yilei Li, Huimei Li, Zhifang Fu. **Jiangsu Provincial CDC:** Ming Wu, Yonglin Zhou, Jinyi Zhou, Ran Tao, Jie Yang, Jian Su. **Suzhou CDC:** Fang liu, Jun Zhang, Yihe Hu, Yan Lu, , Liangcai Ma, Aiyu Tang, Shuo Zhang, Jianrong Jin, Jingchao Liu. **Guangxi Provincial CDC:** Zhenzhu Tang, Naying Chen, Ying Huang. **Liuzhou CDC:** Mingqiang Li, Jinhuai Meng, Rong Pan, Qilian Jiang, Jian Lan,Yun Liu, Liuping Wei, Liyuan Zhou, Ningyu Chen Ping Wang, Fanwen Meng, Yulu Qin,, Sisi Wang. **Sichuan Provincial CDC:** Xianping Wu, Ningmei Zhang, Xiaofang Chen,Weiwei Zhou. **Pengzhou CDC:** Guojin Luo, Jianguo Li, Xiaofang Chen, Xunfu Zhong, Jiaqiu Liu, Qiang Sun. **Gansu Provincial CDC:** Pengfei Ge, Xiaolan Ren, Caixia Dong. **Maiji CDC:** Hui Zhang, Enke Mao, Xiaoping Wang, Tao Wang, Xi zhang. **Henan Provincial CDC:** Ding Zhang, Gang Zhou, Shixian Feng, Liang Chang, Lei Fan. **Huixian CDC:** Yulian Gao, Tianyou He, Huarong Sun, Pan He, Chen Hu, Xukui Zhang, Huifang Wu, Pan He. **Zhejiang Provincial CDC:** Min Yu, Ruying Hu, Hao Wang. Tongxiang CDC: Yijian Qian, Chunmei Wang, Kaixu Xie, Lingli Chen, Yidan Zhang, Dongxia Pan, Qijun Gu. **Hunan Provincial CDC:** Yuelong Huang, Biyun Chen, Li Yin, Huilin Liu, Zhongxi Fu, Qiaohua Xu. **Liuyang CDC:** Xin Xu, Hao Zhang, Huajun Long, Xianzhi Li, Libo Zhang, Zhe Qiu.

Table S1 List of 25 variables included in the FI

No.	Definition according to baseline self-report and/or measurement	Coding of variables
1	Self-reported doctor diagnosis of hypertension, self-reported use of antihypertension drugs, measured SBP ≥140 mmHg, or measured DBP ≥90 mmHg	Yes = 1, No = 0
2	Self-reported doctor diagnosis of heart disease	Yes = 1, No = 0
3	Self-reported doctor diagnosis of stroke or transient ischemic attack	Yes = 1, No = 0
4	Self-reported doctor diagnosis of emphysema or chronic bronchitis	Yes = 1, No = 0
5	Self-reported doctor diagnosis of tuberculosis	Yes = 1, No = 0
6	Self-reported doctor diagnosis of asthma	Yes = 1, No = 0
7	Self-reported doctor diagnosis of peptic ulcer	Yes = 1, No = 0
8	Self-reported doctor diagnosis of gallstone disease, with or without cholecystitis complication	Yes = 1, No = 0
9	Self-reported doctor diagnosis of rheumatoid arthritis	Yes = 1, No = 0
10	Self-reported doctor diagnosis of fracture	Yes = 1, No = 0
11	Self-reported doctor diagnosis of neurasthenia	Yes = 1, No = 0
12	Self-reported doctor diagnosis of diabetes, measured fasting blood glucose ≥7.0 mmol/L, or measured random blood glucose ≥11.1 mmol/L	Yes = 1, No = 0
13	Self-reported doctor diagnosis of cancer	Yes = 1, No = 0
14	Self-reported doctor diagnosis of chronic kidney disease	Yes = 1, No = 0
15	If you were walking on level ground with other healthy people of the same age, would you usually become short of breath or slow down due to chest discomfort?	Yes = 1, No = 0
16	During the past month, did you have any of the following for ≥3 days each week: (1) taking >30 minutes to fall asleep after going bed or waking up in the middle of the night; (2) waking up early and not being able to go back to sleep; or (3) having difficulty staying alert while at work, eating or meeting people during daytime?	Yes = 1, No = 0
17	How often do you have bowel movements each week?	Less than 3 times a week = 1, Others = 0

No.	Definition according to baseline self-report and/or measurement	Coding of variables
18	During the past 12 months, did you have a pain or discomfort in your body lasting ≥3 months that interfered with your life?	Yes = 1, No = 0
19	During the past 12 months, have you usually coughed frequently?	Yes, for ≥3months = 1,<br>Yes, for <3months = 0.5,<br>No = 0
20	Brush teeth rarely or never, or have false teeth	Yes = 1, No = 0
21	During the past 12 months, have you lost weight ≥2.5 kg and not tried to reduce weight intentionally?	Yes = 1, No = 0
22	During the past 12 months, did you feel much more sad, or depressed than usual for two or more weeks?	Yes = 1, No = 0
23	How is your current general health status?	Poor = 1,<br>Fair = 0.5,<br>Good = 0.25,<br>Excellent = 0
24	Measured heart rate	<60 or >100 = 1,<br>≥60 and ≤100 = 0
25	The ratio of measured forced expiratory volume in 1 s over measured forced vital capacity (FEV_1/FVC) <0.7	Yes = 1, No = 0

Table S2 ICD-10 codes used to define the occurrence of disease from baseline to the 2nd resurvey

Diseases	ICD-10 codes
Hypertension	I10-I15
Heart disease	I20-I25
Stroke	I60-I61, I63-I64
Emphysema or chronic bronchitis	J41-J44
Tuberculosis	A15-A19.9, B90-B90.9, J65, K23.0, K67.3, K93.0, M01.1, M49.0, M90.0, N33.0, N74.0, N74.1, O98.0, P37.0, U84.3
Asthma	J45-J46
Peptic ulcer	K25-K28
Gallstone disease	K80-K81
Rheumatoid arthritis	M05, M06, M45
Fracture	S02, S12, S22, S32, S42, S52, S62, S72, S82, S92, T02, T08, T10, T12, T14.2
Neurasthenia	F48.0
Diabetes	E10-E14
Cancer	C00-C97
Chronic kidney disease	D63.1, E10.2, E11.2, I12-I13.9, N02-N08.8, N15.0, N18-N18.9, Q61-Q62.8, N11

Figure S1 Mean FI and frailty prevalence by age and sex among 25,040 participants

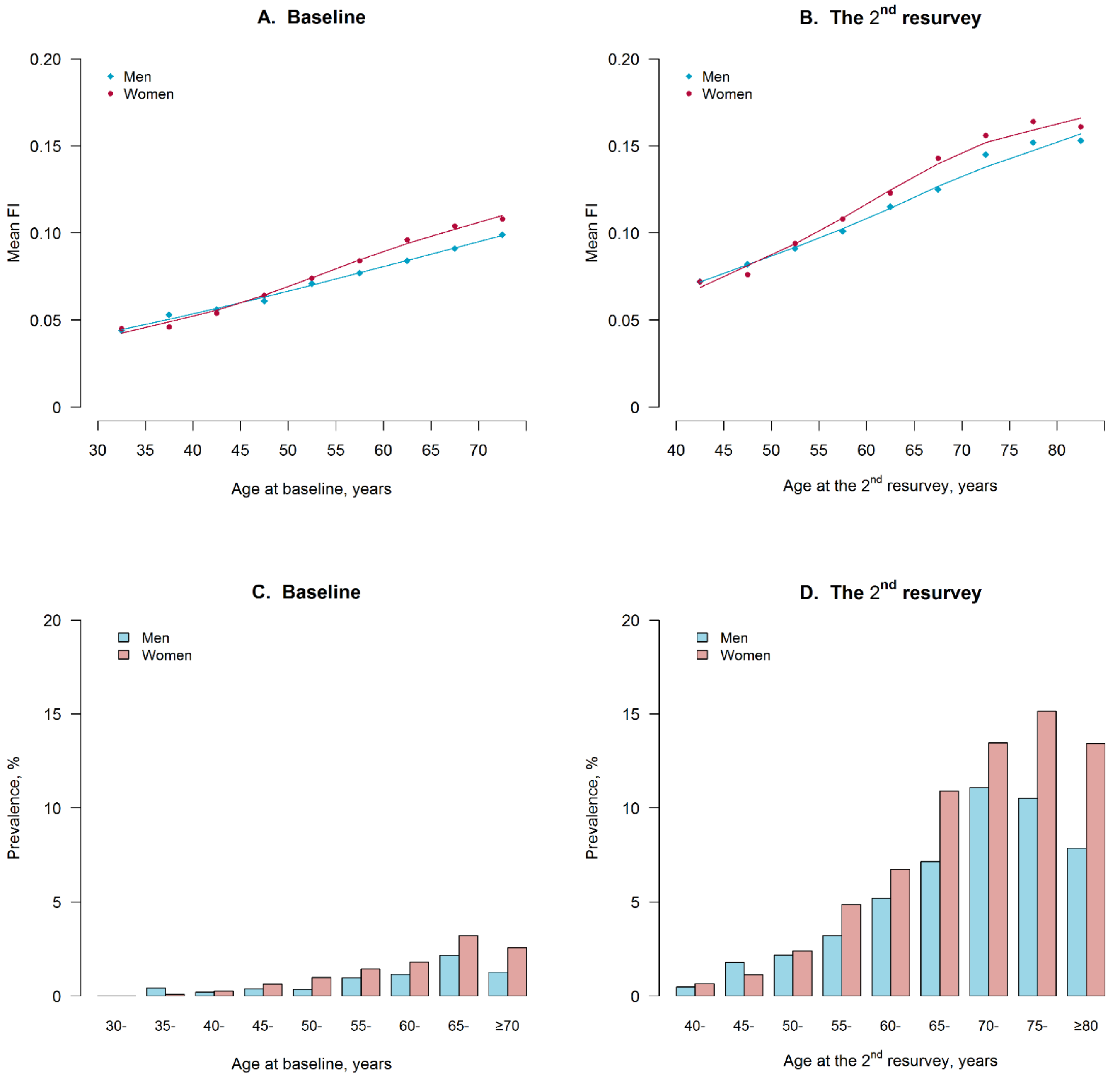

Table S3 Association between FI and total mortality among 25,040 participants

	Death	Death rate (/1000 PYs)	HR (95% CI)
**Frailty status at baseline[a]**			
Robust	448	2.1	1.00
Prefrail	418	4.9	1.40 (1.22-1.61)
Frail	32	11.9	2.58 (1.78-3.75)
Per 0.1 increment of FI			*1.43 (1.29-1.59)*
**Frailty status at the 2nd resurvey[b]**			
Robust	171	3.8	1.00
Prefrail	583	12.7	1.90 (1.59-2.27)
Frail	144	27.2	2.90 (2.28-3.68)
Per 0.1 increment of FI			*1.54 (1.42-1.66)*

Cox models were stratified by age, sex, and study area, with age as the time scale. Multivariable models were adjusted for age (year), education level (no formal school, primary school, middle school, high school, college, or university or higher), smoking status (non-smoker, former smoker who had stopped for reasons other than illness, current smoker or former smoker who had stopped due to illness: 1-14, 15-24, or ≥25 cigarettes or equivalent per day), alcohol consumption (non-drinking, former drinker, current weekly drinker or drinking < 30 g/d of pure alcohol in men or < 15 g/d in women, drinking ≥ 30 g/d of pure alcohol in men or ≥ 15 g/d in women), intake frequency of fresh fruit, vegetables and red meat (days/week: calculated by assigning participants to the midpoint of their consumption category), physical activity (MET-hours/day), body-mass index (kg/m^2), and waist-hip ratio.

[a]The death number refers to death from baseline to the date of December 31, 2017, with the mean follow-up time of 11.8 years.

[b]The death number refers to death from the 2nd resurvey to the date of December 31, 2017, with the mean follow-up time of 3.8 years.

PY = person-year; HR = hazard ratio; CI = confidence interval.

Table S4 Frailty transitions from baseline to the 2nd resurvey

Baseline	The 2nd resurvey			
	Robust	Prefrail	Frail	Total
Robust	9756 (55.2%)	7426 (42.0%)	490 (2.8%)	17672
Prefrail	1808 (25.3%)	4489 (62.9%)	844 (11.8%)	7141
Total	11564	11915	1334	24813

The numbers in brackets indicate the percentage of frailty status at the 2nd resurvey by baseline status.

Table S5 Mean FI value change per 5 years and mean FI value at the 2nd resurvey by baseline frailty status and lifestyle factors

	Baseline robust			Baseline prefrail		
	Number	FI value change per 5 years	Mean FI at 2nd resurvey	Number	FI value change per 5 years	Mean FI at 2nd resurvey
Total	17672	0.034	0.095	7141	0.009	0.151
Smoking status						
Non-smoking	12349	0.032	0.092	5002	0.008	0.148
Former smoker[a]	468	0.036	0.098	183	0.013	0.156
1-14 cig/d	1700	0.035	0.098	722	0.012	0.152
15-24 cig/d	2240	0.037	0.101	840	0.012	0.156
≥25 cig/d	915	0.041	0.108	394	0.015	0.164
Alcohol consumption						
Non-drinking	14460	0.033	0.095	5818	0.009	0.150
Former drinker	509	0.035	0.103	395	0.008	0.158
Weekly or <15/30g/d[b]	1471	0.032	0.094	514	0.010	0.151
≥15/30g/d	1232	0.035	0.099	414	0.013	0.153
Eating fruit and vegetables						
Daily	3140	0.030	0.089	1114	0.007	0.147
Less than daily	14532	0.034	0.096	6027	0.010	0.151
Physical activity (MET-h/day)						
Quartile1 (Lowest)	3653	0.035	0.098	2201	0.011	0.155
Q2	4434	0.033	0.095	1860	0.008	0.149
Q3	4661	0.033	0.094	1639	0.010	0.149
Q4 (Highest)	4924	0.033	0.093	1441	0.009	0.149
BMI (kg/m^2)						
<18.5	658	0.032	0.093	296	0	0.139
18.5-23.9	9383	0.031	0.090	3447	0.007	0.146
24.0-27.9	5880	0.035	0.098	2451	0.011	0.153
≥28.0	1751	0.042	0.112	947	0.017	0.166
WHR						
Men <0.90, women <0.85	8146	0.031	0.089	2723	0.006	0.144
Men 0.90-0.94, women 0.85-0.89	5586	0.034	0.095	2214	0.010	0.149
Men≥0.95, women ≥0.90	3940	0.039	0.106	2204	0.014	0.160

[a]Former smoker refers to those stopped smoking for reasons other than illness. Those who stopped smoking due to illness were classified as daily smoker.
[b]Drinking < 30 g/d of pure alcohol in men or < 15 g/d in women.
The FI value was adjusted for age, sex, and 10 study area.
Abbreviations as in Table 1.

Table S6 Mean FI value change per 5 years and mean FI value at the 2nd resurvey by baseline frailty status and lifestyle change between baseline and the 2nd resurvey

	Baseline robust			Baseline prefrail		
	Number	FI value change per 5 years	Mean FI at 2nd resurvey	Number	FI value change per 5 years	Mean FI at 2nd resurvey
Smoking status						
Constant non-smoking[a]	12553	0.032	0.093	5087	0.008	0.148
Fluctuating status	1194	0.035	0.098	535	0.013	0.157
Desirable transition[b]	930	0.034	0.098	437	0.011	0.154
Undesirable transition[c]	264	0.036	0.097	98	0.022	0.170
Constant smoking	3925	0.038	0.101	1519	0.013	0.157
Alcohol consumption						
Constant non-heavy alcohol drinking[d]	14999	0.033	0.094	5929	0.009	0.149
Fluctuating status	1652	0.036	0.101	765	0.012	0.156
Desirable transition	720	0.035	0.100	362	0.007	0.151
Undesirable transition	932	0.036	0.101	403	0.017	0.161
Constant heavy alcohol drinking	1021	0.036	0.101	447	0.015	0.161
Eating fruit and vegetables						
Constant daily eating	2330	0.028	0.084	795	0.006	0.143
Fluctuating status	4739	0.033	0.095	1695	0.008	0.150
Desirable transition	3929	0.033	0.095	1376	0.008	0.150
Undesirable transition	810	0.035	0.098	319	0.009	0.153
Constant non-daily eating	10603	0.035	0.097	4651	0.011	0.152
Physical activity						
Constant being physically active[e]	6325	0.032	0.091	1747	0.007	0.145
Fluctuating status	6434	0.034	0.095	2545	0.010	0.150
Desirable transition	3174	0.033	0.092	1212	0.009	0.148
Undesirable transition	3260	0.034	0.097	1333	0.011	0.152
Constant low physical activity	4913	0.036	0.100	2849	0.010	0.155
BMI (kg/m^2)						
Constant healthy BMI[f]	7072	0.032	0.090	2572	0.007	0.146
Fluctuating status	3461	0.033	0.095	1392	0.008	0.149
Desirable transition	1150	0.038	0.104	517	0.011	0.155
Undesirable transition	2311	0.030	0.090	875	0.006	0.146
Constant unhealthy BMI	7139	0.036	0.100	3177	0.012	0.155
WHR						
Constant healthy WHR[g]	4454	0.031	0.088	1507	0.006	0.144
Fluctuating status	4947	0.032	0.092	1817	0.007	0.146
Desirable transition	1255	0.035	0.096	601	0.010	0.149
Undesirable transition	3692	0.031	0.090	1216	0.005	0.144
Constant unhealthy WHR	8271	0.036	0.100	3817	0.012	0.155

[a]Non-smoking refers to never smoking or having stopped for reasons other than illness.
[b]Desirable transition refers to transiton from unhealthy lifestyle at baseline to healthy lifestyle at the 2nd resurvey.
[c]Undesirable transition refers to transiton from healthy lifestyle at baseline to unhealthy lifestyle at the 2nd resurvey.
[d]Heavy alcohol drinking refers to drinking ≥30 g/d of pure alcohol in men or ≥15 g/d in women or ex-drinkers.

[e]Being physically active refers to being sex-specific upper 50% of the physical activity level.
[f]Healthy BMI refers to 18.5-23.9 kg/m^2.
[g]Healthy WHR refers to WHR <0.90 in men or <0.85 in women.
The FI value was adjusted for age, sex, and 10 study area.
Abbreviations as in Table 1.

Table S7 Association between baseline lifestyle factors and frailty transitions from baseline to the 2nd resurvey among baseline robust participants by gender

	Men		Women		$P_{interaction}$
	Cases	OR (95% CI)	Cases	OR (95% CI)	
Smoking status[a]					0.003
Non-smoking	727	1.00	4743	1.00	
Former smoker	210	1.10 (0.88-1.38)	15	1.96 (0.71-5.38)	
1-14 cig/d	692	1.17 (1.01-1.36)	97	0.74 (0.54-1.01)	
15-24 cig/d	992	1.35 (1.18-1.55)	-	-	
≥25 cig/d	440	1.56 (1.31-1.86)	-	-	
Alcohol consumption					0.001
Non-drinking	1778	1.00	4716	1.00	
Former drinker	241	1.33 (1.08-1.64)	22	0.48 (0.28-0.81)	
Weekly or <15/30g/d[b]	544	0.89 (0.78-1.03)	69	1.01 (0.71-1.43)	
≥15/30g/d	498	1.01 (0.87-1.18)	48	1.53 (0.97-2.42)	
Eating fruit and vegetables					0.502
Daily	367	1.00	945	1.00	
Less than daily	2694	1.29 (1.10-1.52)	3910	1.19 (1.06-1.34)	
Physical activity (MET-h/day)					0.479
Quartile1 (Lowest)	741	1.06 (0.89-1.25)	1204	1.13 (0.99-1.29)	
Q2	809	0.92 (0.79-1.07)	1274	1.11 (0.98-1.26)	
Q3	756	1.00 (0.87-1.16)	1212	1.04 (0.92-1.16)	
Q4 (Highest)	755	1.00	1165	1.00	
BMI (kg/m^2)					0.745
<18.5	131	1.26 (0.96-1.67)	186	1.10 (0.89-1.36)	
18.5-23.9	1590	1.00	2317	1.00	
24.0-27.9	1026	1.13 (1.00-1.29)	1698	1.13 (1.03-1.24)	
≥28.0	314	1.46 (1.18-1.79)	654	1.59 (1.37-1.85)	
WHR					0.958
Men <0.90, women <0.85	1470	1.00	1799	1.00	
Men 0.90-0.94, women 0.85-0.89	875	1.14 (1.01-1.30)	1642	1.11 (1.00-1.22)	
Men≥0.95, women ≥0.90	716	1.40 (1.20-1.64)	1414	1.31 (1.16-1.47)	

[a]Former smoker refers to those stopped smoking for reasons other than illness. Those who stopped smoking due to illness were classified as daily smoker. For women, we combined 1-14 cig/d, 15-24 cig/d, and ≥25 cig/d into daily smoker, since limited number of daily smokers in women.
[b]Drinking < 30 g/d of pure alcohol in men or < 15 g/d in women.
Multivariable model was adjusted for age, 10 study area, education, and lifestyle factors listed in the table.
Abbreviations as in Table 1.

Table S8 Association of lifestyle changes with frailty transitions from baseline to the 2nd resurvey among baseline robust participants by gender

	Men		Women		$P_{interaction}$
	Cases	OR (95% CI)	Cases	OR (95% CI)	
Smoking status					0.339
Constant non-smoking[a]	834	1.00	4745	1.00	
Fluctuating status	506	1.14 (0.98-1.34)	41	0.66 (0.43-1.03)	
Constant smoking	1721	1.28 (1.14-1.45)	69	0.82 (0.57-1.18)	
Alcohol consumption					0.160
Constant non-heavy alcohol drinking[b]	1965	1.00	4709	1.00	
Fluctuating status	660	1.13 (0.99-1.28)	112	0.86 (0.66-1.13)	
Constant heavy alcohol drinking	436	1.11 (0.95-1.30)	34	1.47 (0.87-2.48)	
Eating fruit and vegetables					0.086
Constant daily eating	236	1.00	713	1.00	
Fluctuating status	761	1.35 (1.10-1.66)	1363	1.28 (1.11-1.47)	
Constant non-daily eating	2064	1.48 (1.20-1.81)	2779	1.30 (1.12-1.50)	
Physical activity					0.650
Constant being physically active[c]	928	1.00	1409	1.00	
Fluctuating status	1065	1.03 (0.91-1.17)	1794	1.14 (1.03-1.26)	
Constant low physical activity	1068	1.13 (0.97-1.31)	1652	1.31 (1.16-1.47)	
BMI (kg/m^2)					0.004
Constant healthy BMI[d]	1208	1.00	1778	1.00	
Fluctuating status	621	1.16 (1.01-1.34)	897	0.96 (0.86-1.07)	
Constant unhealthy BMI	1232	1.11 (0.97-1.27)	2180	1.15 (1.04-1.27)	
WHR					0.059
Constant healthy WHR[e]	949	1.00	854	1.00	
Fluctuating status	825	1.07 (0.94-1.22)	1236	1.00 (0.89-1.13)	
Constant unhealthy WHR	1287	1.30 (1.14-1.49)	2765	1.22 (1.09-1.37)	

[a]Non-smoking refers to never smoking or having stopped for reasons other than illness.
[b]Heavy alcohol drinking refers to drinking ≥30 g/d of pure alcohol in men or ≥15 g/d in women or ex-drinkers.
[c]Being physically active refers to being sex-specific upper 50% of the physical activity level.
[d]Healthy BMI refers to 18.5-23.9 kg/m^2.
[e]Healthy WHR refers to WHR <0.90 in men or <0.85 in women.
Multivariable model was adjusted for age, 10 study area, education, and lifestyle changes listed in the table.
Abbreviations as in Table 1.

Table S9 Association between baseline lifestyle factors and frailty transitions by not including follow-up information to supplement disease status at the second resurvey

	Robust worsening		Prefrail worsening	
	Cases	OR (95% CI)	Cases	OR (95% CI)
Smoking status				
Non-smoking	5104	1.00	488	1.00
Former smoker[a]	213	1.12 (0.90-1.38)	27	1.92 (1.17-3.15)
1-14 cig/d	716	1.09 (0.95-1.24)	69	1.19 (0.85-1.65)
15-24 cig/d	939	1.27 (1.11-1.44)	63	1.26 (0.87-1.80)
≥25 cig/d	412	1.46 (1.23-1.73)	30	1.39 (0.88-2.22)
Alcohol consumption				
Non-drinking	6058	1.00	577	1.00
Former drinker	246	1.14 (0.94-1.38)	33	0.85 (0.57-1.26)
Weekly or <15/30g/d[b]	573	0.93 (0.82-1.05)	44	0.96 (0.67-1.37)
≥15/30g/d	507	1.01 (0.88-1.17)	23	0.71 (0.44-1.14)
Eating fruit and vegetables				
Daily	1228	1.00	136	1.00
Less than daily	6156	1.22 (1.11-1.34)	541	1.15 (0.91-1.46)
Physical activity (MET-h/day)				
Quartile1 (Lowest)	1819	1.10 (0.99-1.22)	278	1.15 (0.86-1.54)
Q2	1962	1.05 (0.95-1.16)	179	0.94 (0.70-1.27)
Q3	1838	1.03 (0.94-1.12)	135	1.14 (0.85-1.52)
Q4 (Highest)	1765	1.00	85	1.00
BMI (kg/m^2)				
<18.5	297	1.18 (1.00-1.39)	22	0.84 (0.53-1.34)
18.5-23.9	3636	1.00	283	1.00
24.0-27.9	2530	1.10 (1.02-1.19)	258	1.15 (0.94-1.40)
≥28.0	921	1.53 (1.36-1.73)	114	1.14 (0.87-1.49)
WHR				
Men <0.90, women <0.85	3029	1.00	222	1.00
Men 0.90-0.94, women 0.85-0.89	2366	1.14 (1.06-1.23)	201	1.01 (0.81-1.25)
Men≥0.95, women ≥0.90	1989	1.34 (1.22-1.47)	254	1.23 (0.98-1.55)

[a]Former smoker refers to those stopped smoking for reasons other than illness. Those who stopped smoking due to illness were classified as daily smoker.

[b]Drinking < 30 g/d of pure alcohol in men or < 15 g/d in women.

Multivariable model was adjusted for age, sex, 10 study area, education, and lifestyle factors listed in the table.

Abbreviations as in Table 1.

Table S10 Association between lifestyle changes and frailty transitions by not including follow-up information to supplement disease status at the second resurvey

	Robust worsening		Prefrail worsening	
	Cases	OR (95% CI)	Cases	OR (95% CI)
Smoking status				
Constant non-smoking[a]	5211	1.00	499	1.00
Fluctuating status	495	0.99 (0.86-1.15)	56	1.25 (0.88-1.78)
Constant smoking	1678	1.21 (1.09-1.36)	122	1.17 (0.87-1.57)
Alcohol consumption				
Constant non-heavy alcohol drinking[b]	6220	1.00	578	1.00
Fluctuating status	724	1.09 (0.97-1.22)	65	1.00 (0.74-1.35)
Constant heavy alcohol drinking	440	1.10 (0.95-1.27)	34	1.05 (0.71-1.57)
Eating fruit and vegetables				
Constant daily eating	880	1.00	97	1.00
Fluctuating status	1991	1.32 (1.18-1.48)	193	1.31 (0.99-1.73)
Constant non-daily eating	4513	1.38 (1.23-1.55)	387	1.21 (0.91-1.62)
Physical activity				
Constant being physically active[c]	2177	1.00	101	1.00
Fluctuating status	2662	1.09 (1.01-1.17)	224	1.09 (0.84-1.42)
Constant low physical activity	2545	1.22 (1.11-1.33)	352	1.14 (0.87-1.50)
BMI (kg/m^2)				
Constant healthy BMI[d]	2764	1.00	208	1.00
Fluctuating status	1442	1.08 (0.99-1.18)	128	1.06 (0.83-1.34)
Constant unhealthy BMI	3178	1.12 (1.04-1.21)	341	1.14 (0.93-1.40)
WHR				
Constant healthy WHR[e]	1679	1.00	125	1.00
Fluctuating status	1912	1.02 (0.94-1.12)	156	1.02 (0.78-1.32)
Constant unhealthy WHR	3793	1.26 (1.15-1.37)	396	1.18 (0.93-1.50)

[a]Non-smoking refers to never smoking or having stopped for reasons other than illness.
[b]Heavy alcohol drinking refers to drinking ≥30 g/d of pure alcohol in men or ≥15 g/d in women or ex-drinkers.
[c]Being physically active refers to being sex-specific upper 50% of the physical activity level.
[d]Healthy BMI refers to 18.5-23.9 kg/m^2.
[e]Healthy WHR refers to WHR <0.90 in men or <0.85 in women.
Multivariable model was adjusted for age, sex, 10 study area, education, and lifestyle changes listed in the table.
Abbreviations as in Table 1.

Table S11 Association of baseline lifestyle factors with prefrail regression and prefrail worsening

	Prefrail regression		Prefrail worsening	
	**Cases**	**OR (95% CI)**	**Cases**	**OR (95% CI)**
Smoking status				
Non-smoking	1270	1.00	600	1.00
Former smoker[a]	43	1.24 (0.83-1.87)	30	1.86 (1.16-3.00)
1-14 cig/d	173	1.03 (0.82-1.31)	91	1.32 (0.97-1.79)
15-24 cig/d	219	0.93 (0.74-1.18)	84	1.38 (0.99-1.91)
≥25 cig/d	103	0.92 (0.68-1.24)	39	1.44 (0.94-2.19)
Alcohol consumption				
Non-drinking	1468	1.00	717	1.00
Former drinker	87	0.84 (0.64-1.11)	46	0.90 (0.63-1.28)
Weekly or <15/30g/d[b]	143	1.05 (0.83-1.33)	49	0.84 (0.59-1.18)
≥15/30g/d	110	0.81 (0.62-1.05)	32	0.73 (0.48-1.10)
Eating fruit and vegetables				
Daily	242	1.00	155	1.00
Less than daily	1566	0.93 (0.77-1.12)	689	1.21 (0.97-1.52)
Physical activity (MET-h/day)				
Quartile1 (Lowest)	381	0.84 (0.70-1.01)	335	1.02 (0.78-1.33)
Q2	459	1.10 (0.92-1.30)	227	0.94 (0.72-1.24)
Q3	498	1.08 (0.92-1.27)	170	1.11 (0.85-1.45)
Q4 (Highest)	470	1.00	112	1.00
BMI (kg/m^2)				
<18.5	84	1.13 (0.85-1.50)	30	0.89 (0.59-1.35)
18.5-23.9	970	1.00	354	1.00
24.0-27.9	576	0.88 (0.77-1.01)	317	1.15 (0.96-1.38)
≥28.0	178	0.73 (0.59-0.91)	143	1.18 (0.92-1.51)
WHR				
Men <0.90, women <0.85	788	1.00	278	1.00
Men 0.90-0.94, women 0.85-0.89	564	0.89 (0.77-1.02)	247	0.96 (0.79-1.18)
Men≥0.95, women ≥0.90	456	0.79 (0.67-0.93)	319	1.15 (0.93-1.42)

[a]Former smoker refers to those stopped smoking for reasons other than illness. Those who stopped smoking due to illness were classified as daily smoker.
[b]Drinking < 30 g/d of pure alcohol in men or < 15 g/d in women.
Multivariable model was adjusted for age, sex, 10 study area, education, and lifestyle factors listed in the table. The reference group was those remain prefrail.

Table S12 Association of lifestyle changes with prefrail regression and prefrail worsening

	Prefrail regression		Prefrail worsening	
	**Cases**	**OR (95% CI)**	**Cases**	**OR (95% CI)**
Smoking status				
Constant non-smoking[a]	1292	1.00	613	1.00
Fluctuating status	123	0.96 (0.74-1.25)	75	1.40 (1.02-1.92)
Constant smoking	393	0.92 (0.75-1.12)	156	1.20 (0.91-1.58)
Alcohol consumption				
Constant non-heavy alcohol drinking[b]	1509	1.00	713	1.00
Fluctuating status	194	0.96 (0.78-1.17)	85	1.03 (0.79-1.36)
Constant heavy alcohol drinking	105	0.78 (0.60-1.01)	46	1.08 (0.75-1.54)
Eating fruit and vegetables				
Constant daily eating	173	1.00	111	1.00
Fluctuating status	414	0.86 (0.68-1.08)	223	1.26 (0.96-1.65)
Constant non-daily eating	1221	0.84 (0.67-1.05)	510	1.29 (0.98-1.70)
Physical activity				
Constant being physically active[c]	611	1.00	127	1.00
Fluctuating status	689	0.98 (0.85-1.13)	287	1.10 (0.87-1.39)
Constant low physical activity	508	0.84 (0.71-0.99)	430	1.04 (0.81-1.33)
BMI (kg/m^2)				
Constant healthy BMI[d]	722	1.00	262	1.00
Fluctuating status	365	0.98 (0.84-1.15)	163	1.09 (0.88-1.35)
Constant unhealthy BMI	721	0.87 (0.76-1.00)	419	1.13 (0.94-1.37)
WHR				
Constant healthy WHR[e]	430	1.00	151	1.00
Fluctuating status	483	0.92 (0.78-1.08)	200	1.02 (0.81-1.29)
Constant unhealthy WHR	895	0.82 (0.70-0.96)	493	1.14 (0.92-1.42)

[a]Non-smoking refers to never smoking or having stopped for reasons other than illness.
[b]Heavy alcohol drinking refers to drinking ≥30 g/d of pure alcohol in men or ≥15 g/d in women or ex-drinkers.
[c]Being physically active refers to being sex-specific upper 50% of the physical activity level.
[d]Healthy BMI refers to 18.5-23.9 kg/m^2.
[e]Healthy WHR refers to WHR <0.90 in men or <0.85 in women.
Multivariable model was adjusted for age, sex, 10 study area, education, and lifestyle changes listed in the table.
The reference group was those remain prefrail.
